# The Evolving Role of Living Donor Liver Transplantation in the Management of Colorectal Liver Metastases

**DOI:** 10.3390/curroncol33030171

**Published:** 2026-03-16

**Authors:** Abu Bakar Hafeez Bhatti, Muhammad Nauman-ul-Haq, Muslim Atiq, Usman Shafiq Khokhar, Azhar Shafi

**Affiliations:** 1 Department of HPB Surgery and Liver Transplantation, Shifa International Hospital, Islamabad 44000, Pakistan; naumanulhaq23@gmail.com (M.N.-u.-H.); drusmanshafique11@gmail.com (U.S.K.); 2 Department of Surgery, Shifa Tameer-e-Millat University, Islamabad 44000, Pakistan; 3 Department of Gastroenterology and Hepatology, Shifa International Hospital, Islamabad 44000, Pakistan; muslim.atiq@shifa.com.pk; 4 Department of Medical Oncology, Shifa International Hospital, Islamabad 44000, Pakistan; azhar.shafi@shifa.com.pk

**Keywords:** colorectal liver metastases, chemotherapy, deceased donor liver transplantation, hepatectomy, living donor liver transplantation

## Abstract

Surgical resection remains the cornerstone of curative-intent therapy for colorectal liver metastases (CRLM). However, a substantial proportion of patients present with technically unresectable diseases or develop intrahepatic recurrence, despite optimal multimodal treatment. In this setting, liver transplantation (LT) has emerged as a potential strategy for durable, cancer-free survival in selected patients. Early experience with deceased donor liver transplantation (DDLT) for CRLM showed encouraging outcomes, but broader implementation has been constrained by ethical concerns and logistical barriers related to organ scarcity. Living donor liver transplantation (LDLT) offers a fundamentally different paradigm, potentially mitigating these limitations while introducing distinct technical, ethical, and logistical considerations. In this manuscript, we delineate the technical and logistic differences between DDLT and LDLT in the context of CRLM and highlight advantages unique to LDLT-based strategies. We contrast historical data with emerging contemporary evidence, with particular emphasis on LDLT outcomes. We critically examine evolving patient selection frameworks, incorporating molecular profiling and circulating tumor DNA-based liquid biopsy. In addition, we report our institutional experience with left lobe LDLT for CRLM in a patient who remains disease-free 30 months after transplantation. We conclude with a comprehensive appraisal of the current LDLT literature in CRLM and propose directions for future research.

## Simple Summary

Surgical resection offers the best chance of cure for patients with advanced colorectal cancer with liver involvement. Unfortunately, surgical resection is not feasible in many patients due to the extent of liver involvement. In recent years, liver transplantation has emerged as an alternative treatment option in selected patients not suitable for resection. Early results using livers from deceased donors are encouraging but organ shortage remains the most significant challenge. Living donor liver transplantation might overcome many obstacles posed by deceased donation, but not without its own unique ethical and medical considerations. In this review, we discuss the key differences between deceased and living donor liver transplantation for colorectal cancer, summarize current global evidence, and report a successful case from out center. We also highlight novel investigations and future directions that will likely define the role of liver transplantation for colorectal cancer in the future.

## 1. Historical Context of Liver Transplantation for Colorectal Liver Metastases

### 1.1. Early Experience

Colorectal cancer (CRC) is the third most frequently diagnosed cancer globally. Liver is the most common site of involvement, with synchronous liver metastases in approximately 25% and metachronous lesions in 50% patients [1]. Despite multimodality treatment involving complex resections and ablations, close to 80% of colorectal liver metastases (CRLM) remain unresectable. This is attributed to the extent of bilateral disease, inadequate future liver remnant (FLR), and proximity to critical vessels [2,3]. For these patients, indefinite use of systemic chemotherapy might offer palliation but little chance for long-term cure. Liver transplantation (LT) is a radical but potentially curative option where total hepatectomy removes all known intrahepatic tumor burden [4]. The initial experiences of LT for CRLM had mixed outcomes. In fact, in the 1980s and 1990s, LT for CRLM was performed without stringent selection criteria, and was associated with early and widespread recurrence, leading to clinical abandonment of the concept [5].

### 1.2. The Paradigm Shift: The SECA Trials

The Norwegian SECA (SEcondary CAncer) trials explored the feasibility of LT in CRLM [6]. The outcomes were dramatically superior, with a 5-year overall survival (OS) of 60% compared with a 5-year OS < 10% with historic cohorts treated with chemotherapy alone. These results not only provided the proof of concept that LT was potentially curative in CRLM but also identified certain negative prognostic factors, which led to the development of the OSLO score, which forms the basis for candidate selection today. Not long after, the enthusiasm for LT in CRLM highlighted a critical ethical challenge: the routine allocation of scarce DDLT grafts to expanding oncological indications meant higher competition for donor organs for otherwise standard indications. This led to the exploration of alternate sources like living donor liver transplantation (LDLT) for CRLM [7].

## 2. Rationale for LDLT in CRLM

The application of LDLT in the CRLM setting is driven by specific advantages that address the logistical and biological constraints inherent to DDLT.

### 2.1. Resolution of the Allocation Conflict

In LDLT, the graft is procured from a designated living donor outside the queue, and therefore, organs are not diverted from patients waiting for LT for non-oncological life-threatening indications (e.g., acute liver failure, decompensated liver disease). As long as the donor risk is kept minimal, LDLT for CRLM is ethically defensible, since the principle of justice in organ allocation is not compromised [8,9].

### 2.2. Improved Timing

Patients eligible for LT for CRLM usually undergo neoadjuvant chemotherapy to reduce tumor burden and determine tumor biology. Patients with progressive disease despite chemotherapy are considered to have aggressive biology and are excluded [10,11]. When LT is indicated, LDLT offers a distinct advantage of precisely timing the transplant. In DDLT, the waiting time can be unpredictable, and the disease might progress beyond what is considered acceptable at some point, becoming unsuitable for LT. LDLT allows the transplant to be scheduled during the period of maximal stable response to neoadjuvant therapy, often referred to as the “window of stability.” This ensures that patient undergoing LDLT has favorable tumor kinetics at the time of transplantation [9,11].

### 2.3. Superior Graft Quality and Minimal Ischemia

Living donors are carefully selected after extensive workup and investigations. Only healthy and voluntary liver donors with pristine livers are considered eligible for donation. The grafts procured from these donors usually have minimal steatosis and short cold ischemia times (CIT), usually under one hour [12]. In contrast, CIT can extend to many hours (6–12+) in DDLT. Prolonged CIT is associated with reperfusion injury and non-anastomotic biliary strictures, the major cause of post-transplant morbidity [13,14].

## 3. LDLT vs. DDLT: Technical, Logistical, and Ethical Dichotomy

While the total hepatectomy procedure remains constant, the technical and logistical differences between LDLT and DDLT are profound and impact decision-making for CRLM (Table 1).

### 3.1. Technical Complexity and Graft Selection in LDLT

LDLT is technically far more complex than standard DDLT. The donor hepatectomy must be meticulously performed to ensure both donor safety and optimal graft function. The most common grafts used for adult CRLM are the right lobe or modified right lobe grafts [12].

• Right Lobe Graft: Provides sufficient volume for the adult recipient but poses the highest risk to the donor where more than 60% of the total liver is donated.
• Left Lobe/Left Lateral Grafts: Typically reserved for smaller recipients.
• Dual Grafts (Rare): Technically very challenging and involve procurement of two grafts from two living donors to meet graft volume requirements of the recipient. Performed in selected centers worldwide and remains controversial.

In LDLT, high hilar dissection is performed during the recipient operation to facilitate complex biliary and vascular reconstruction with the partial graft. Hepatic venous outflow reconstruction is a critical determinant of graft function, particularly with right lobe grafts, where large-caliber anastomosis of the right hepatic vein and reconstruction of significant segment V and VIII veins using venous conduits may be required to prevent venous congestion and ensure adequate drainage. Portal vein reconstruction must ensure unobstructed inflow while avoiding excessive portal hyper perfusion, which can contribute to small-for-size syndrome (SFSS). Moreover, variations in donor portal anatomy pose a unique challenge during reconstruction and may require back table portoplasty and extension grafts. Hepatic arterial reconstruction is typically performed under loupe or microscopic magnification, given the small caliber of the vessels and the potentially devastating consequences of hepatic artery thrombosis. Specifically, in patients with prior transarterial treatments for CRLM, hepatic artery integrity might be questionable. In exclusive LDLT systems, salvage options in cases of arterial dissection are limited and may involve the use of saphenous vein conduits or synthetic grafts [15,16]. Biliary reconstruction may also be technically demanding, particularly in right lobe grafts where multiple small bile ducts are common; these may require ductoplasty, multiple duct-to-duct anastomoses or Roux-en-Y hepaticojejunostomy to minimize the risk of postoperative leaks and strictures.

The risk of Small-for-Size Syndrome (SFSS), where the transplanted partial graft is too small relative to the recipient’s body mass, is a critical concern, necessitating precise preoperative volumetric assessment using computed tomography (CT) or magnetic resonance imaging (MRI), and represents an important factor for potential donor refusal [13,14,17,18,19]. In selected cases, inflow modulation strategies such as splenic artery ligation or splenectomy may be considered to mitigate excessive portal inflow and reduce the risk of graft dysfunction.

### 3.2. The Donor Safety and Ethical Imperative

Donor safety is the absolute ethical priority in LDLT. For CRLM, the donor is subjected to major surgery for the benefit of a recipient who has metastatic cancer, a disease with an inherent risk of recurrence, even after LT [20]. This places a significant burden on the transplant team to ensure that donor mortality is maintained at <0.5% and the donor understands the recipient’s prognosis, high recurrence risk, and the overall risks associated with donor and recipient surgery. This demanding ethical framework necessitates highly centralized expertise and strict adherence to established protocols.

### 3.3. Prior Interventions

Patients with CRLM often present with a liver previously injured by long courses of highly effective, but toxic, chemotherapy (e.g., oxaliplatin-induced sinusoidal obstruction syndrome, irinotecan-induced steatohepatitis), prior resection, or ablation. Such interventions increase intraoperative bleeding risk and may complicate recipient hepatectomy [21]. The use of a partial, rather than whole, graft LT must be weighed against this background pathology.

## 4. The Critical Role of Evolving Patient Selection Criteria

The success of LT for CRLM relies heavily on patient selection. As the field matures, the selection criteria have slowly moved away from using anatomical metrics alone to sophisticated molecular profiling.

### 4.1. Foundational Biological Criteria (SECA)

In the context of LT for CRLM, the SECA criteria defined the favorable phenotype. Sustained response to chemotherapy before LT was considered mandatory and tumor progression despite chemotherapy was considered a signal of aggressive tumor biology. A significant reduction or normalization of CEA with chemotherapy was indicative of favorable biology and some protocols use a CEA level < 80 ng/mL for inclusion. A disease-free interval of >12 months between primary tumor resection and LT was considered favorable. Extrahepatic disease was considered a contraindication for LT unless enrolled in expanded clinical trials [6,22].

### 4.2. The Integration of Molecular Profiling

Certain molecular signatures are stronger predictors of systemic recurrence than conventional radiologic criteria.

*Rat Sarcoma (RAS) and B-Rapidly Accelerated Fibrosarcoma (BRAF):* RAS [Kirsten RAS (KRAS), Neuroblastoma RAS (NRAS)] mutations are common in metastatic CRC and have important prognostic implications. RAS mutations are associated with reduced response to anti-Epidermal Growth Factor Receptor (EGFR) therapies and often correlate with more aggressive tumor biology compared with RAS wild-type tumors, which tend to respond better to EGFR-targeted agents. BRAFV600E mutation is universally recognized as a major negative prognostic factor and often serves as an exclusion criterion due to rapid progression and high recurrence risk [23,24].

*Microsatellite Instability (MSI) and Mismatch Repair (MMR) Status:* While MSI-High tumors often have better overall prognosis in early stages, their role in the transplant setting is complex and under active investigation [23,25,26].

### 4.3. Liquid Biopsy and Circulating Tumor (ct)DNA

The use of ctDNA offers a dynamic, non-invasive assessment of Minimal Residual Disease (MRD). In pre-transplant settings, ctDNA can detect occult disease missed on conventional imaging (PET, CT). Persistent ctDNA after neoadjuvant chemotherapy is indicative of micrometastases and these patients are at high risk of recurrence post-LT. After LT, ctDNA negativity is representative of tumor eradication and its reappearance, which can occur months before radiological recurrence and is a marker of disease relapse, allows pre-emptive escalation of systemic therapy [27,28].

## 5. Clinical Trials Landscape

**Figure 1:**
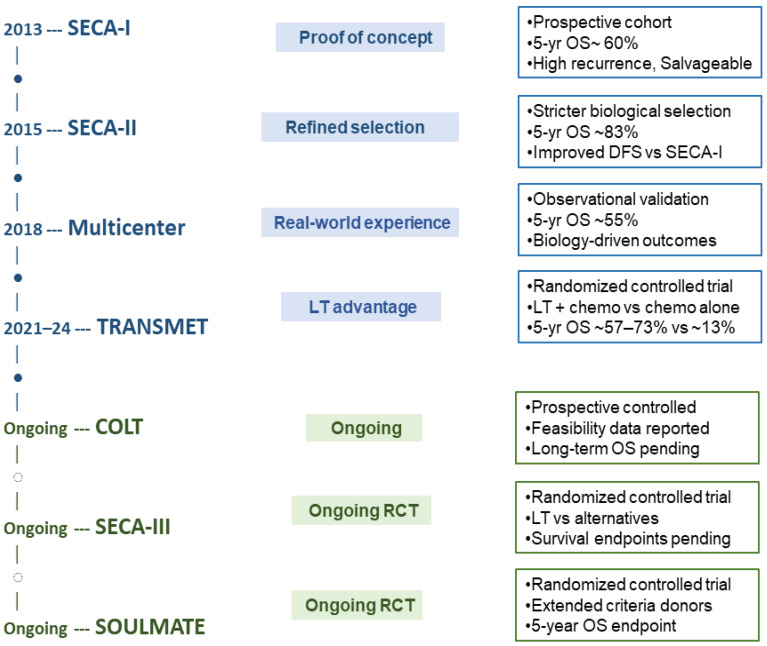
Clinical trial landscape for LT in CRLM.

The field of LT for CRLM is generally defined by the SECA single-arm trials and prospective registries, with various Randomized Controlled Trials (RCTs) underway (Figure 1).

### 5.1. Major DDLT Trials for CRLM

The SECA-I trial enrolled patients with liver-only, unresectable CRLM, prior R0 resection of the primary tumor, absence of extrahepatic disease, and at least six weeks of systemic chemotherapy, without strict limits on tumor burden or CEA levels. The 5-year OS was 60% and the recurrence rate was high but amenable to salvage treatment [6]. SECA-II introduced more stringent criteria, including radiographic response to chemotherapy, specified minimum intervals from diagnosis to transplant, and tighter limits on tumor size and burden, achieving improved long-term outcomes [22]. SECA-III, an ongoing randomized trial, aims to explore the benefits of LT under broad inclusion criteria, comparing LT to contemporary oncologic therapies [29]. The recently published TRANSMET trial required ECOG performance status 0–1, controlled disease (stable or partial response) by RECIST, absence of extrahepatic spread, and exclusion of high-risk molecular subtypes (BRAFV600E), and CEA level cutoffs to optimize transplant benefit [2]. The multicenter randomized trial SOULMATE has a similar selection policy as TRANSMET but limits tumor size to 10 cm, has more rigorous high-risk molecular subtype classification, and uses extended-criteria donor grafts. The COLT trial is prospectively enrolling hyper selected patients with unresectable liver-only CRLM who are RAS and BRAF wild-type and microsatellite-stable, with CEA levels < 50 ng/mL, demonstrating objective response to first-/second-line chemotherapy sustained for ≥4 months, and a maximum of two prior chemotherapy lines, with unresectability confirmed by multidisciplinary review using technical and tumor burden criteria [30]. In addition, a number of other trials are evaluating the role of LT in CRLM and are nearing completion [29].

### 5.2. The Absence of LDLT Randomized Trials

No RCTs currently exist comparing LDLT versus DDLT or LDLT versus standard-of-care resection/chemotherapy for CRLM. Randomizing patients to a potentially curative therapy (LT) versus standard palliative care (chemotherapy) is difficult once the efficacy of LT is established in selected patients. Furthermore, randomizing a healthy donor’s participation in LDLT is ethically impossible. The highly selective nature of the patient cohort, the heterogeneity of protocols across centers, and the inherent difficulty in standardizing surgical techniques across institutions make RCTs logistically prohibitive [11,31].

## 6. Case Presentation

### 6.1. Initial Presentation and Management

In March 2020, a 41-year-old female presented to the emergency department with acute-onset right hypochondrium pain and localized tenderness. Imaging identified a subcapsular hepatic hematoma originating from an underlying liver lesion. Hemostasis was achieved via urgent trans arterial embolization (TAE) and subsequent ultrasound-guided biopsy confirmed metastatic adenocarcinoma. A staging PET-CT and upper and lower gastrointestinal endoscopy localized the primary lesion in the descending colon.

### 6.2. Neoadjuvant Phase and Primary Resections

**Figure 2:**
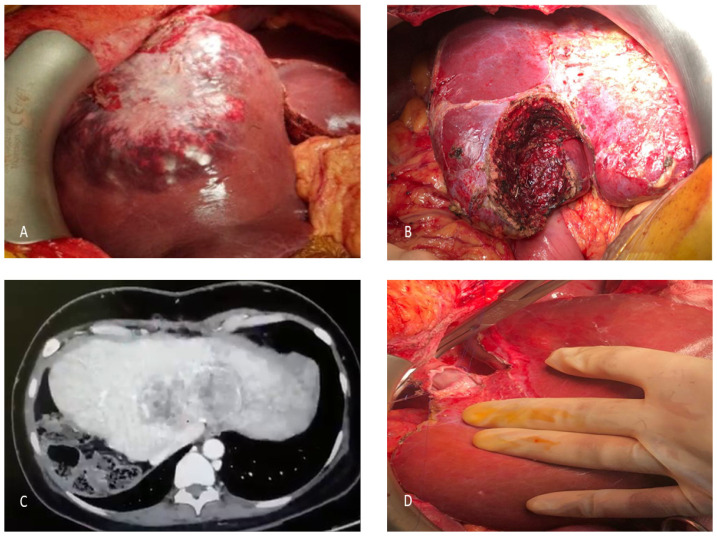
(**A**) Intraoperative photo showing large right lobe CRLM planned for right hepatectomy. (**B**) Intraoperative photo of segment IVB resection for recurrent CRLM. (**C**) Large unresectable recurrence close to the middle and left hepatic veins. (**D**) Hepatic vein anastomoses in left lobe LDLT.

The patient commenced upfront systemic chemotherapy (Avastin and Oxaliplatin). Following four cycles, a significant biochemical response was noted, with CEA levels decreasing from >1000 ng/mL to 42 ng/mL. Imaging confirmed a partial response per modified RECIST criteria. In August 2020, following MDT review, she underwent a right hepatectomy with negative margins (Figure 2A). This was followed by a left hemicolectomy in October 2020; histopathology revealed a well-differentiated adenocarcinoma (pT2N1) with clear surgical margins.

### 6.3. Recurrence and Pregnancy

In June 2021, surveillance imaging detected two new lesions in segment 4, managed with wide local excision (Figure 2B). Shortly thereafter, the patient became pregnant. During the gestation period, systemic therapy was deferred; she successfully delivered a healthy neonate and remained clinically stable. However, in June 2022, follow-up imaging revealed recurrent intrahepatic metastases. Due to the proximity of the lesions to the left and middle hepatic veins, the disease was deemed surgically unresectable (Figure 2C). The patient resumed aggressive chemotherapy (Oxaliplatin, 5-FU, and Cetuximab, followed by Irinotecan/Cetuximab). By May 2023, repeat CT imaging demonstrated partial radiological response in the hepatic lesions.

### 6.4. LDLT

In light of her favorable prognostic indicators including young age, absence of extrahepatic disease, negative genetic mutations (KRAS/BRAF wild-type), and prolonged chemo-sensitivity (20 cycles of chemotherapy), the patient was counseled for LDLT as a salvage curative intent therapy. The patient’s husband volunteered as the only ABO-compatible donor. Anatomical variations in the donor required complex surgical planning, including the harvest of a left lobe graft with the Middle Hepatic Vein (MHV) (Figure 2D). The use of the right hepatic lobe would have required reconstruction of the right hepatic vein, three inferior right hepatic veins, and segment V and VIII veins. Challenges included a replaced left hepatic artery (LHA) arising from the left gastric artery and an early extra-hepatic origin of the segment IV artery. The transplant was performed in May 2023 with a GRWR of 0.93. The patient had an uneventful postoperative course and was discharged on 12th postoperative day. She developed biliary anastomotic stricture, which was successfully managed with endoscopic biliary stenting. Her CEA level remains within normal range, and surveillance CT scans remain negative for recurrence 30 months after left lobe LDLT.

## 7. Global LDLT Experience

The average waiting time for LT in Norway is less than a month. In 2020 alone, 1105 patients in the United States died on the waiting list, compared to only four in Norway [6,22,32,33]. Therefore, organ shortage represents a critical challenge in LT for CRLM and highlights the need for exploring LDLT as a viable option. To the best of our knowledge, a total of 62 patients with CRLM have undergone LDLT (Table 2). Graft type was not reported in 10 patients. In the remaining 52 patients, 40 patients received right lobe, seven patients received left lateral segment and five patients received left lobe graft. No major donor morbidity was reported.

### 7.1. Early Experience with LDLT

In 2006, Kocman and colleagues successfully performed LDLT in a 46-year-old female, after chemotherapy and multiple hepatectomies, who remained alive and recurrence-free at five years post-LT [34]. Toso and colleagues performed LT in 12 patients, including one LDLT, and concluded that long-term survival without recurrence was achievable in highly selected cases [35]. Konigsrainer and colleagues published the first case report of partial resection and segment 2–3 transplantation with delayed total hepatectomy (RAPID procedure) from a living donor [36]. The patient underwent right hemicolectomy, left hepatectomy, and segment 2–3 implantation simultaneously. At five months post-transplant, micrometastases were detected in bone and lung, prompting adjuvant chemotherapy; the patient was alive at 22 months post-transplant.

### 7.2. Recent Reports on LDLT for CRLM

More recent reports have continued to demonstrate the potential role of LDLT in highly selected patients. Lerut and colleagues reported outcomes of one right lobe and one left lobe graft LDLT, where a DFS of 4 months and 32 months, and overall survival of 28 and 32 months was achieved, respectively, with both patients alive at last follow-up [38]. Choi and colleagues reported DFS and OS of 13 years in a patient who underwent right lobe LDLT for life-threatening bleeding from esophageal varices and liver cirrhosis after seven cycles of hepatic arterial infusion chemotherapy, two years after resection of primary colonic tumor [39]. LDLT for CRLM after pulmonary metastatectomy has also been reported with OS greater than 24 months [45].

### 7.3. Selection Criteria in LDLT

**Figure 3:**
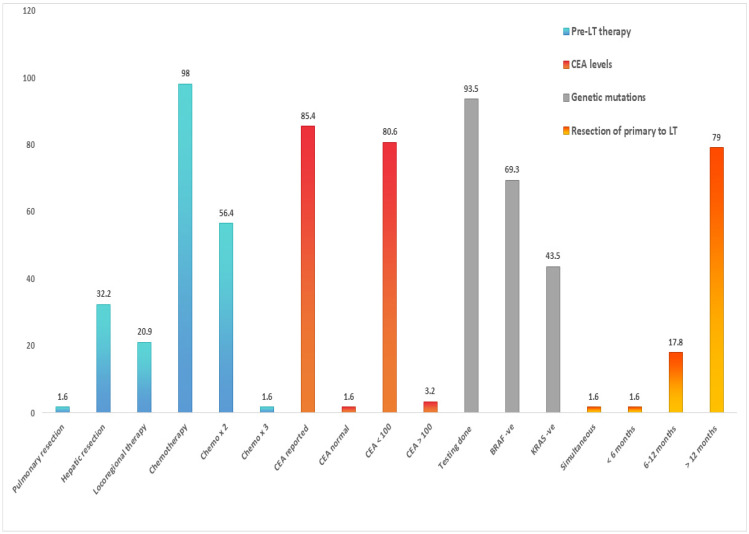
Percentage of patients assessed for clinically relevant pre-transplant factors before undergoing LDLT for CRLM worldwide.

The selection criteria for LDLT in the context of CRLM are not universally standardized. However, several common themes emerge across various centers and guidelines. These include considerations for pre-transplant therapies, serum CEA levels, the results of genetic testing, the time elapsed between diagnosis and LT, and the utilization of neoadjuvant chemotherapy (Figure 3).

The Toronto group refined selection criteria for LT by incorporating biological and radiological parameters. Patient selection was based on response to chemotherapy, ≤T4a lesion, >6 months of chemotherapy, time from primary resection to transplant ≥ 6 months, stable or decreasing CEA levels, and stability or regression of liver metastasis ≥ 3 months before screening and until transplant (Supplementary Table S1). They reported a 3-year OS of 100% and DFS of 68% at median follow-up of 14.8 months in seven patients [41] (Table 3). The Rochester group explored the feasibility of LDLT in CRLM, adopting SECA and IHPBA guidelines. In 23 patients, 3-year OS of 91% and DFS of 40% were reported with a median follow-up of 541 days, suggesting that LDLT was feasible in well-selected patients [46]. In a single-center North American study, ten patients with CRLM underwent LDLT. Although graft types were not documented, most patients received prior systemic therapy, with a significant portion also undergoing liver-directed treatments such as hepatic artery infusion and resection. The mean DFS was 2.2 years, while the mean OS reached 3.0 years, with 90% patients alive at the last follow-up [44].

Among the included studies reporting recurrence patterns, pulmonary metastases were the most common site of recurrence, occurring in 19.3% of patients. Hepatic recurrence was observed in 4.8%, while bone metastases and intra-abdominal nodal recurrence were each reported in 3.2% of patients. Adrenal and locoregional recurrences were less frequent, each occurring in 1.6% of cases. Recurrence at multiple sites was documented in 3.2% of patients. Post-transplant recurrence was primarily managed with systemic chemotherapy. In selected cases, surgical or locoregional interventions were undertaken, including pulmonary metastasectomy (lung segmentectomy), adrenalectomy for adrenal metastases, and radiofrequency ablation for isolated hepatic recurrence.

### 7.4. Novel Techniques in LDLT

After the introduction of DD-RAPID concept in 2015 in CRLM, the first formal report describing the LD-RAPID procedure of left lateral segment LDLT was published in 2019 [36,48]. This concept gained popularity and was widely adopted by clinical trials like LIVER-T(W)O-HEAL study. This protocol started in March 2018 in Germany and includes patients with <T3N1 and stable disease after at least 8 weeks of chemotherapy. This trial is ongoing, and to date, 7 patients have been transplanted. One patient developed early tumor recurrence and died 24 months after LT. One patient died at 4 months due to lung embolism and three patients are alive and tumor-free after an observation time of 6 to 18 months. Two patients underwent left lobe graft, and their outcome has not been reported.

### 7.5. Immunosuppression

While tumor biology remains the primary determinant of post-transplant outcomes, pharmacologic immunosuppression may influence the growth of residual micrometastatic disease after transplantation. Calcineurin inhibitors (CNIs) can impair immune surveillance against circulating tumor cells and experimental data suggest they may also promote tumor progression through upregulation of transforming growth factor-β (TGF-β) and vascular endothelial growth factor (VEGF), thereby enhancing angiogenesis, invasiveness, and metastatic potential. These mechanisms may contribute to earlier recurrence in the post-transplant setting [49]. In contrast, mammalian target of rapamycin (mTOR) inhibitors possess antiproliferative and anti-angiogenic properties and may attenuate tumor progression [50]. Early clinical experience in LT for CRLM, including the Norwegian SECA studies, incorporated sirolimus-based immunosuppression [6]. Although robust prospective data specific to CRLM remain limited, there is evidence for reduced recurrence after LT for HCC, where mTOR-based regimens and CNI minimization have been implemented. Therefore, early CNI minimization and consideration of mTOR-based protocols represent an important adjunct in reducing recurrence in LDLT for CRLM [51].

### 7.6. Role of Systemic Therapy

There is not a uniformly accepted, standardized neoadjuvant chemotherapy regimen specifically for LT in CRLM. Published protocols and consensus guidance from recent literature and selection criteria incorporate systemic chemotherapy as a prerequisite before LT, with some reasonably consistent elements [52,53], such as at least 6 months of pre-transplant chemotherapy with documented response or disease stabilization on imaging (RECIST criteria) before transplantation. The chemotherapy regimens include oxaliplatin-based and irinotecan-based doublet or triplet combinations such as FOLFOX (5-FU, leucovorin, oxaliplatin), FOLFIRI (5-FU, leucovorin, irinotecan), CAPOX/XELOX (capecitabine, oxaliplatin), and FOLFOXIRI (5-FU, leucovorin, oxaliplatin, irinotecan). Response assessment every 2–3 months is typically recommended, and stable disease or partial response (e.g., ≥30% reduction by RECIST) during chemotherapy forms part of many LT selection criteria. Similarly, there is no universally mandated number of cycles before LT, but sufficient duration must be allowed to demonstrate sustained disease control (often ≥6 months total).

With regard to adjuvant chemotherapy after LT for CRLM, there is no standard regimen, and it is used only in a minority of patients. When used, it is typically started one month after LT and continued for six months of tolerated [54]. Therefore, the safety profile, specifically in the context of post-transplant immunosuppression, remains questionable. Importantly, commonly used cytotoxic agents such as oxaliplatin and irinotecan are known to cause sinusoidal injury and steatohepatitis, raising concern for hepatotoxicity and graft dysfunction [55].

## 8. The Way Forward

The overall outcomes of DDLT for CRLM in the United States have been somewhat suboptimal, with graft quality likely playing a major role. The use of marginal grafts, many with underlying fibrosis, combined with higher MELD scores reflecting extensive prior treatment, may contribute to increased short-term mortality [56]. Importantly, underlying fibrosis has been associated with higher recurrence rates after resection, highlighting its potential impact on post-transplant outcomes [57]. At the same time, accumulating evidence suggests that carefully selected CRLM patients can benefit from transplantation, prompting the introduction of MELD exception points to provide equitable access without disadvantaging other patients [58]. These observations underscore the rationale for LDLT in CRLM, though success will depend on strict adherence to high procedural standards, integration of predictive models to refine patient selection, and exploration of neoadjuvant strategies to optimize outcomes.

### 8.1. Standardizing LDLT Protocols

For patients with CRLM who do not have portal hypertension or cirrhosis, the usual graft requirements may not always apply. However, the field still lacks prospective validation for lower GRWR thresholds. Centers looking to safely implement LDLT should start by clearly defining minimum graft volumes tailored to the recipient’s body mass and by establishing perioperative protocols to minimize biliary complications and graft dysfunction [59,60]. Equally important is a systematic approach to donor evaluation, including psychological assessment, cardiopulmonary screening, and structured long-term follow-up to ensure donor well-being [61]. By building these safeguards into routine practice, LDLT can be delivered more safely and reproducibly.

### 8.2. Predictive Modeling for Patient Selection

Current criteria for selecting CRLM patients are overly simplistic, relying on cutoffs that fail to capture tumor biology and recurrence risk [22]. Moving forward, centers should adopt multivariable predictive models that integrate clinical, pathological, and molecular factors [62,63]. Emerging biomarkers such as ctDNA, RAS/BRAF mutation status, MSI, and PET metabolic response can be incorporated to refine risk stratification. Using these tools, clinicians can identify patients who are likely to benefit most from transplantation and tailor follow-up and adjuvant strategies to those at higher risk.

### 8.3. Neoadjuvant and Pre-Transplant Strategies

Neoadjuvant therapies can help control micrometastatic disease before transplantation, though evidence in CRLM is still limited [64,65,66]. In practice, patients should receive standard systemic chemotherapy (for example, FOLFOX or FOLFIRI) for a defined period, typically around six months, with documented disease stabilization or response. For MSI-H/dMMR tumors, immunotherapy may be considered in clinical trial settings, but careful attention must be paid to timing relative to transplantation to avoid immunologic complications. Close monitoring with imaging and biomarkers is essential to guide treatment decisions and optimize outcomes.

### 8.4. Ethical Considerations and Donor Protection

The ethical stakes in LDLT for CRLM are particularly high because donors are often close relatives, and the recipient’s prognosis may be uncertain. It is vital to ensure that donors are truly volunteering and fully understand the risks, not just of the surgery, but also the limited and uncertain benefit to the recipient [61]. Counseling should include clear discussion of recurrence risk and potential need for further therapy [56,57]. Independent ethics committees should review each case to confirm that the procedure is justified, that the donor’s autonomy is protected, and that institutional safeguards are in place. Finally, donors should be followed long-term, both for physical recovery and psychosocial well-being, to ensure that donation does not result in harm.

## 9. Conclusions

LDLT represents a critical and expanding therapeutic modality for CRLM. It bypasses organ allocation challenges, enables timely transplantation during the window of maximal response, and provides grafts with excellent quality. While robust RCTs comparing LDLT with other treatments are absent due to ethical constraints, high-quality data from international registries have established its role in CRLM. The future success of LDLT relies on refinements in selection criteria, adherence to standardized protocols, and ensuring long-term donor safety.

## Figures and Tables

**Table 1:** Key differences between LDLT and DDLT in the context of CRLM.

Feature	LDLT	DDLT
Organ Source	Partial liver from a healthy and voluntary living donor	Whole or partial liver from a deceased donor
Allocation System	Bypasses the MELD score Usually center-specific	Based on MELD score Requires exception points for oncological indications
Timing	Elective After documented response to neoadjuvant treatment Usually during the window of maximal response	Non-elective Waiting time can be unpredictable and prolonged
Cold Ischemia Time (CIT)	Minimal (typically <1 h) Superior initial graft function Reduced risk of non-anastomotic biliary strictures	Variable and often prolonged (6–12+ h) Higher risk of reperfusion injury
Ethical Burden	High ethical burden involving surgical risk to the donor	Ethical conflict regarding organ diversion from non-oncological patients
Surgical Complexity	High technical complexity due to partial graft, complex vascular, and biliary reconstruction	Lower complexity, especially with whole-organ transplantation

**Table 2:** Global LDLT experience for CLRM.

Author	Cases	Year	Country	Primary	Pre-Transplant Treatment	CEA Level ng/mL	Genetic Analysis
Kocman B [34]	1 RLG	2011	Croatia	Left colon	Chemotherapy Hepatectomy	N/A	N/A
Toso C [35]	1 RLG	2017	Switzerland	N/A	N/A	N/A	N/A
Konigsrainer A [36]	1 LLSG	2018	Germany	Right colon	Chemotherapy LD-RAPID	61	Absent KRASG12V, TP53, and ERBB2 mutations
Fernandes ESM [37]	1.RLG	2019	Brazil	Left colon	Hepatectomy Chemotherapy	3.8	Absent K-RAS mutation
Lerut J [38]	1 RLG 1 LLG	2019	Belgium	Transverse colon 1 Sigmoid colon 1	Hepatectomy	<100	Absent KRAS, BRAF, MMR
Choi J U [39]	1 RLG	2020	Korea	Left colon	7 sessions of HAI therapy	220	N/A
Nadalin S [40]	6 LLSG 2 LLG	2020	Germany 5 Italy 2 Belgium 1	N/A	Chemotherapy	N/A	N/A
Rajendran L [41]	6 RLG 1 LLG	2023	Canada	Left Colon 5 Right Colon 1 Rectum 1	Chemotherapy Hepatectomy in 2 HAIP in 3	Downward trend	Absent BRAF V600E mutation
Fernandes EDSM [42]	4 RLG	2023	Brazil		Hepatectomy Radiofrequency ablation Chemotherapy Transarterial chemoembolization	(8.3–181)	Absent K-RAS mutation
Kotenco OH [43]	1 LLG	2023	Ukraine	Left Colon	Chemotherapy	<10	N/A
Kaltenmeier C [44]	10	2024	USA	Right Colon 2 Transverse Colon 1 Left Colon 3 Rectum 4	Chemotherapy Targeted therapy Hepatectomy HAI RFA	<100	Absent BRAF mutation
Alshamrani A [45]	1 RLG 1 LLG	2024	Korea	Left Colon	Chemotherapy Hepatectomy RFA Pulmonary metastatectomy	N/A	N/A
Byrne MM [46]	23 RLG	2025	USA	Left 15 Right 2 Rectum 6	Chemotherapy HAI Hepatectomy MWA RFA SIRT	<80	Absent BRAF V600E mutations and/or high MSI. Right-sided primary tumors and patients with KRAS and TP53 mutations required an observation time of 18 months.
Lucchese AM [47]	1 RLG	2025	Brazil	Left Colon	Chemotherapy	3.2	Absent BRAF and KRAS mutation
Current report	1 LLG	2026	Pakistan	Left colon	Hepatectomy Chemotherapy	<5	Absent KRAS and BRAF V600 mutation

HAI = hepatic artery infusion; HAIP = hepatic artery infusion pump; MWA = microwave ablation; N/A = not available; RFA = radiofrequency ablation; SIRT = selective internal radiation therapy; ERBB2 = erb-b2 receptor tyrosine kinase 2.

**Table 3:** Outcomes in different LDLT studies for CRLM.

Author	Recurrence	Treatment of Recurrence	Follow-Up	RFS	OS
Kocman B [34] *N* = 1	N/A	N/A	5 years	5 years	5 years
Toso C [35] *N* = 1	Lung-5 Liver-3 Peritoneum-1	Chemotherapy plus radiation	Median-26 (0–108) months	1 year 56% 3 year 38% 5 year 38%	1 year 83% 3 year 62% 5 year 50%
Konigsrainer A [36] *N* = 1	Bone + Lung (5 months)	Chemotherapy + Radiation	22 months	5 months	22 months
Fernandes ESM [37] *N* = 1	No	No	2 months	2 months	2 months
Lerut J [38] *N* = 2	Lung mets-1 (4 months)	Resection + Chemotherapy	28 months 32 months	4 months 32 months	28 months 32 months
Choi J U [39] *N* = 1	No	No	13 Years	13 Years	13 Years
Nadalin S [40] *N* = 8	1/5	Not reported	6–18 months	Not reported	Not reported
Rajendran L [41] *N* = 7	Lung-1 (3.3 months) Intra-abdominal node-1 (12.4 months)	Chemotherapy	Median-14.8 months	85% at 1 year 68% at 3 year	100% at 1 year 100% at 3 year
Fernandes EDSM [42] *N* = 4	Liver + lung + bone Adrenal gland No No	Chemotherapy-1 Adrenalectomy with chemotherapy-1 N/A No	3 years 2.5 years 1 year <1 year	14 months 14 months 1 year <1 year	3 years 2.5 years 1 year <1 year
Kotenco OH [43] *N* = 1	Lung 1 (11 months)	Lung resection + Chemotherapy	13 months	11 months	13 months
Kaltenmeier C [44] *N* = 10	Liver-1 (422 days) Lung-1 (55 days) Celiac axis +lung-1 (155 days)	RFA and radiation-1 Lung segmentectomy-1 Lung segmentectomy + adjuvant chemotherapy-1	Median (1.5 Years)	62% at median follow-up of 1.5 year 2.2 years mean	100% at median follow-up of 1.5 years 3 years mean
Alshamrani A [45] *N* = 2	Lung-1 (4 months)	Resection + Chemotherapy	28 months 32 months	4 months 32 months	28 months 32 months
Byrne MM [46] *N* = 23	Locoregional-1 Liver-1 Lung-4	Lung metastatectomy in one patient	541 Days (179–998)	100% at 1 year 40% at 3 year	100% at 1 year 91% at 3 year
Lucchese A M [47] *N* = 1	No	No	10 months	10 months	10 months
Current report *N* = 1	No	No	32 months	32 months	32 months

## Data Availability

The data is available from the corresponding author upon reasonable request.

## Supplementary Materials

The following supporting information can be downloaded at https://www.mdpi.com/article/10.3390/curroncol33030171/s1, Table S1: Previously reported Inclusion and exclusion criteria for LDLT in CRLM [41,44,46].

## Abbreviations

The following abbreviations are used in this manuscript:

CEA	Carcinoembryonic Antigen
CLRM	Colorectal Liver Metastases
ctDNA	Circulating Tumor Deoxyribo Nucleic Acid
DDLT	Deceased Donor Liver Transplantation
LDLT	Living Donor Liver transplantation
SECA	Secondary Cancer
