# Supplementary material for: The Evolving Role of Living Donor Liver Transplantation in the Management of Colorectal Liver Metastases
Source: Curr Oncol. 2026 Mar 16;33(3):171. doi: 10.3390/curroncol33030171 (PMC13025921; doi:10.3390/curroncol33030171)
Supplement: Supplementary file 1 [file curroncol-33-00171-s001.zip › curroncol-4150737-supplementary.pdf]

Supplementary Table S1. Previously reported Inclusion and exclusion criteria for LDLT in CRLM.

Author	Inclusion Criteria	Exclusion criteria
Kaltenmeier C [42]	Histologically confirmed adenocarcinoma of the colon/rectum, resected with adequate margins (at least 2 cm for rectal carcinoma).<br><br>Histologically confirmed CRLM.<br><br>LT to be considered at least 6 months after diagnosis/ resection of primary tumor.<br><br>Received at least 6 to 12 weeks of CT with no evidence of disease progression.<br><br>No signs of local recurrence on colonoscopy within the past 6 to 12 mo before LT evaluation.<br><br>No signs of local or extrahepatic metastases on CT CAP/MRI/ PET CT/Bone scan at time of LT evaluation.<br><br>CEA < 100 ng/dL at time of LT evaluation.<br><br>No limit in terms of number or size of lesions.<br><br>(ECOG) performance status: 0-1<br><br>Available living donor.	Poor performance status.<br><br>Diagnosis of other malignancy as outlined in University of Pittsburgh Medical Center (UPMC) guidelines: "Liver Transplantation in Patients with a History of Cancer".<br><br>Previous or current extrahepatic metastases or local recurrence.<br><br>Any general contraindication for LT.<br><br>Proto-oncogene BRAF mutant tumors.
Byrne M M [41]	Histologically confirmed CRC adenocarcinoma with CRLM.<br><br>No extrahepatic metastatic disease or local recurrence.<br><br>No signs of primary tumor recurrence or extrahepatic metastases on CT or MRI and whole-body PET-CT at least 6 weeks before LDLT.<br><br>No signs of local recurrence on colonoscopy, performed within 12 months before LDLT.<br><br>1 year or more from CRLM diagnosis.<br><br>Six months or more from primary CRC resection.<br><br>Disease response or stability in the last 6 months before LDLT.	BRAF V600E.<br><br>High microsatellite instability.<br><br>Perihepatic or retroperitoneal malignant lymph node positivity at time of diagnostic laparotomy.<br><br>Exclusion for CEA >80 µg/L at time of listing.<br><br>Exclusion for disease progression at time of LDLT.<br><br>Right-sided primary tumors and patients with combined KRAS and TP53 mutations require an observation period of 18 months or longer from CRLM diagnosis.

	ECOG:0-1<br><br>Oslo score ≤1<br><br>No upper limit on size and number of lesions.<br><br>Stable or partial response on standard oncologic treatment in the 6 months before LDLT.<br><br>If CEA is <80 µg/dL but is rising at time of transplant, LDLT is delayed for further oncologic workup.	
**Rajendran L [40]**	Age 18–68 y at study entry.<br><br>ECOG score <2 at all times before LDLT.<br><br>Proven CRLM, isolated to the liver.<br><br>Bilobar and unresectable liver metastases (on consensus), with no major vascular invasion.<br><br>Primary CRC tumor stage ≤T4a.<br><br>Time from primary resection to transplant ≥6 months<br><br>Undergone ≥6 mo of systemic CT (eg FOLFOX/ FOLFIRI ± biologic) or agreeable to restart and be maintained on CT until dropout or transplant.<br><br>Stability or regression of liver metastases ≥3 months before screening and until transplant CEA levels stable or decreasing at all points before transplant.<br><br>At least 1 "acceptable" ABO-compatible living-donor candidate<br><br>Negative serum pregnancy test (for women of childbearing potential).<br><br>Use of adequate barrier birth control in both men and women during course of trial.<br><br>Willing and able to provide informed consent.	Progression of liver metastases at any point before transplant.<br><br>BRAF-V600E primary tumor.<br>CEA levels >100 at final pre-transplant assessment.<br><br>Radiographic large vessel invasion.<br>Previous lung resection.<br><br>Previous or concurrent cancer within 5 y.<br><br>Previous history of solid organ transplantation.<br><br>Pulmonary insufficiency, history of cardiac disease, renal dysfunction (creatinine clearance l <50 mL/min).<br><br>Debilitating neuropathy (CTCAE >grade 2).<br><br>Uncontrolled infection, known history of HIV infection, or chronic HBV or HCV infection.<br><br>Substance abuse, psychological or social conditions that interfere with study participation.<br><br>Known or suspected allergy to any agent given within the trial.<br><br>Pregnant or breastfeeding patients.

		Any unstable condition that could jeopardize patient health, safety, and study compliance.
